# Prognostic Features and Potential for Immune Therapy in Metastatic Mismatch Repair‐Deficient Colorectal Cancer: A Retrospective Analysis of a Large Consecutive Population‐Based Patient Series

**DOI:** 10.1002/cam4.70555

**Published:** 2025-01-09

**Authors:** Erkki‐Ville Wirta, Hanna Elomaa, Jukka‐Pekka Mecklin, Toni T. Seppälä, Marja Hyöty, Jan Böhm, Maarit Ahtiainen, Juha P. Väyrynen

**Affiliations:** ^1^ Department of Gastroenterology and Alimentary Tract Surgery Tampere University Hospital Tampere Finland; ^2^ Faculty of Medicine and Health Technology Tampere University and Tays Cancer Center, Tampere University Hospital Tampere Finland; ^3^ Department of Biological and Environmental Science University of Jyväskylä Jyväskylä Finland; ^4^ Department of Education and Research The Wellbeing Services of Central Finland Jyväskylä Finland; ^5^ Faculty of Sport and Health Sciences University of Jyväskylä Jyväskylä Finland; ^6^ Department of Gastrointestinal Surgery Helsinki University Central Hospital, University of Helsinki Helsinki Finland; ^7^ Applied Tumor Genomics, Research Program Unit University of Helsinki Helsinki Finland; ^8^ Department of Pathology Wellbeing Services County of Central Finland Jyväskylä Finland; ^9^ Translational Medicine Research Unit, Medical Research Center Oulu Oulu University Hospital, and University of Oulu Oulu Finland

## Abstract

**Background:**

Immune checkpoint inhibition therapies have provided remarkable results in numerous metastatic cancers, including mismatch repair–deficient (dMMR) colorectal cancer (CRC). To evaluate the potential for PD‐1 blockade therapy in a large population‐based cohort, we analyzed the tumor microenvironment and reviewed the clinical data and actualized treatment of all dMMR CRCs in Central Finland province between 2000 and 2015.

**Material and Methods:**

Of 1343 CRC patients, 171 dMMR tumors were identified through immunohistochemical screening. Histological tumor parameters were evaluated from hematoxylin‐ and eosin‐stained whole‐slide samples. CD3 and CD8 immunohistochemistry were analyzed to calculate T‐cell densities in the tumor center and invasive margin, and G‐cross function values to estimate cancer cell–T‐cell co‐localization. Multiplex immunohistochemistry was used to identify CD68+PD‐L1+ and CD3+PD‐1+ immune cells and PD‐L1 expression on tumor cells.

**Results:**

A total of 35 (20%) patients with dMMR tumors were diagnosed as having a metastatic disease. Twelve patients (34%) were fit enough to be offered oncological treatments at the onset of non‐curable metastatic disease. High proportions of necrosis and stroma were common in metastatic tumors and were associated with worse survival. Crohn's‐like reaction, T‐cell proximity score, and CD68+/PD‐L1+ on the tumor center and invasive margin were independent prognostic immune factors.

**Conclusion:**

As dMMR CRC patients are generally older, with often significant comorbidities, only a limited portion of patients with metastatic dMMR tumors ended up in oncological treatments. Many of the metastatic tumors presented features that may impair response to PD‐1 blockade therapy.

## Graphical abstract

Mismatch repair–deficient colorectal cancer patients are generally older, with often significant comorbidities, and only a limited portion of patients with metastatic tumors underwent oncological treatments. Many of the metastatic tumors present features that may impair response to PD‐1 blockade therapy. High proportions of necrosis and stroma were common in metastatic tumors and were associated with worse survival; however, high Crohn's‐like reaction density, T‐cell proximity score, and CD68+/PD‐L1+ cell number in the tumor center and invasive margin were independent prognostic immune factors for improved survival.

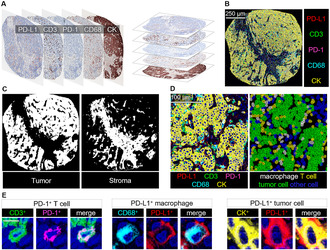

## 1 Background

Microsatellite instability (MSI) is caused by a deficiency in the DNA mismatch repair system (MMR) and is seen in about 15% of colorectal cancers (CRCs). Most MSI CRCs are sporadic, usually due to an epigenetic inactivation of the *MLH1* gene through promoter hypermethylation. About 15%–20% of MSI CRCs are of hereditary origin and are associated with Lynch syndrome, with a germline mutation in one of the MMR genes *MLH1*, *MSH2*, *MSH6*, or *PMS2*, or an alteration in the *EPCAM* gene that causes the silencing of *MSH2* [1, 2]. Sporadic MSI CRCs are associated with female gender, older age, and location in the right hemicolon, while hereditary MSI CRCs occur at a younger age and are less gender‐ or location‐specific [3].

MSI tumors are hypermutated and express abundant neoantigens, explaining their tendency to exhibit a high infiltration of lymphocytes and frequent peritumoral tertiary lymphoid structures referred to as Crohn's‐like lymphoid reaction (CLR), often with an earlier stage occurrence and improved prognosis [4, 5, 6]. Even though metastatic MSI CRCs comprise only about 3%–5% of metastasized CRCs, these tumors are associated with chemoresistance to standard treatment and a dismal disease outcome [7, 8].

As a sign of striving for immune evasion in a hostile microenvironment, MSI tumors frequently express checkpoint inhibition proteins, for example, PD‐L1 (programmed death‐ligand 1) [9]. Consequently, new immunotherapeutic approaches through cancer immune checkpoint blockade have proved effective in numerous cancers, including MMR‐deficient CRC. Recently, pembrolizumab, a PD‐1 (programmed cell death protein‐1) inhibitor, was approved for the first‐line treatment of patients with unresectable or metastatic MMR‐deficient CRC by the US Food and Drug Administration, soon followed by the European Medicine Evaluation Agency [10].

In this study, we reviewed the clinical data and actualized treatment of all identified MMR‐deficient CRCs belonging to the population‐based series of all consecutive CRCs diagnosed in the province of Central Finland (population of 270,000) during 2000–2015. We aimed to assess the differences of the tumor characteristics of metastatic compared to non‐metastatic cases and to evaluate the prognostic features of the tumor microenvironment. We focused on the characteristics of metastatic cases that could potentially influence response to immune checkpoint therapy which was not available during their treatment period. We used detailed methods to evaluate the composition of the tumor microenvironment, including CLR, infiltrating CD3+ and CD8+ lymphocytes, and the expression of checkpoint inhibition pathway proteins PD‐1/PD‐L1 on CD3 + lymphocytes, CD68+ macrophages, and tumor cells.

## 2 Methods

### 2.1 Patients

**FIGURE 1:**
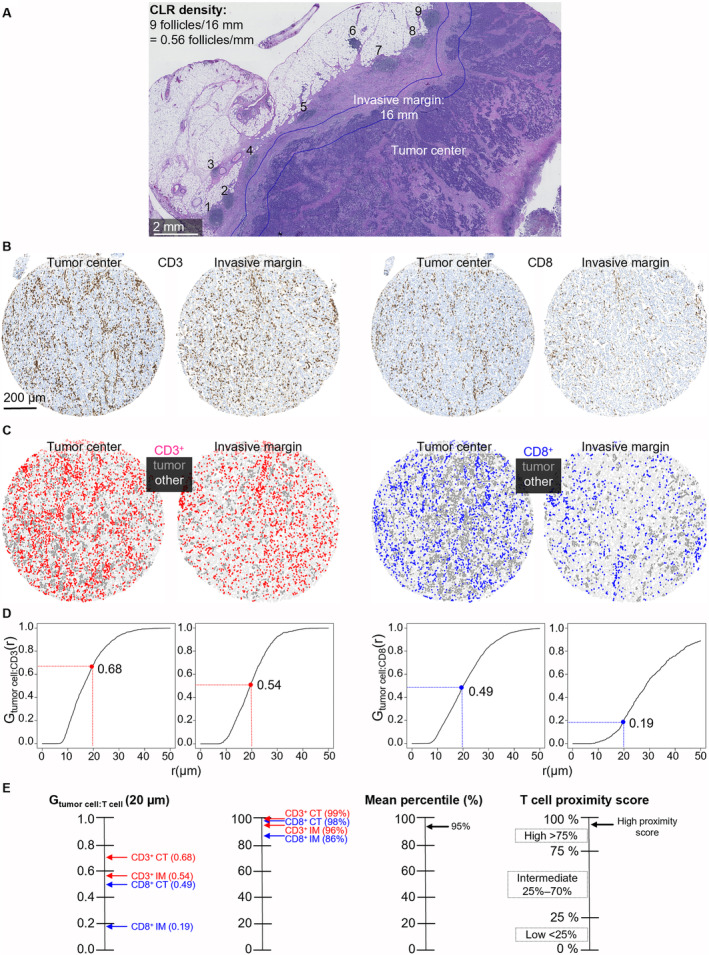
Crohn's‐like reaction density and T‐cell proximity score analysis. (A) Hematoxylin‐ and eosin‐stained whole‐slide image representing Crohn's‐like reaction density analysis. (B) Examples of CD3 and CD8 immunohistochemistry tissue microarray images from the tumor center and the invasive margin of a single tumor. (C) Corresponding phenotyping maps for T cells, tumor cells, and other cells. (D) G‐cross [G_tumor:T cell_] function curves representing the likelihood of any tumor cell in the tumor core having at least one CD3+/CD8+ T cell within a radius *r*. (E) Calculation chart for T‐cell proximity score.

The study population consisted of 1343 patients with colorectal adenocarcinoma treated at Central Finland Central Hospital between 2000 and 2015 with adequate tumor samples for MMR immunohistochemistry. The age and comorbidity burden (Charlson comorbidity index, CCI) effect on multimodal treatment and survival was recently reported from those with primarily resectable tumors [11]. Also, standard surgical procedures, including exclusion criteria from operative treatment, neoadjuvant and adjuvant treatments, and follow‐up have been reported earlier [11]. Clinical and original histopathological data were retrospectively retrieved from hospital records. All patients in this study underwent surgical treatment of the primary tumor, and all histopathological analyses presented were conducted on the primary tumor samples. The TNM stages are based on pathological assessment performed at the time of treatment. Additional histological tumor parameters (CLR, differentiation according to WHO 2019 criteria, tumor budding according to the International Tumor Budding Consensus Conference, lymphovascular invasion, tumor necrosis and intratumoral stroma) were evaluated by a study pathologist (JPV) from hematoxylin‐ and eosin‐ (H&E‐) stained whole‐tissue section samples. The median was used as a cut‐off point for intratumoral stroma percentage. CLR density was evaluated by calculating the number of CLRs at the tumor invasive margin of H&E‐stained whole‐slide sections and then dividing it by the length of the margin as presented in Figure 1a [12]. The cut‐off value of 0.42/mm for CLR density was obtained from ROC curves drawn in relation to disease‐specific survival (Figure S1). A total of 171 MMR‐deficient tumors were identified and included for further analysis. Of those, 35 (20%) were diagnosed with metastatic disease either during the primary treatment (*n* = 11, 31%) or later during follow‐up (*n* = 24, 69%).

### 2.2 Immunohistochemical Analysis

Two 1‐mm diameter cores from the tumor center and two from the invasive margin of a representative formalin‐fixed paraffin‐embedded tumor sample with the deepest cancer invasion were selected from each tumor to prepare tissue microarray (TMA) blocks. The arrays were constructed using a TMA Master II tissue microarrayer (3DHistech Ltd., Budapest, Hungary) [13]. Staining protocols and antibodies used for the immunohistochemistry of T cells, as well as the multiplex immunohistochemistry used to identify CD68+PD‐L1+, CD3+PD‐1+ immune cells and PD‐L1+ tumor cells, have been described by Elomaa et al. [13, 14] Immunohistochemical screening for DNA mismatch repair (MMR) deficiency with MLH1, MSH2, MSH6, and PMS2 expression and for BRAFV600E mutation status were performed according to Seppälä et al. [13, 15] Seven patients had been identified by germline testing to have Lynch syndrome. All the samples were digitalized with a NanoZoomer‐XR (Hamamatsu Photonics, Hamamatsu City, Japan, resolution 0.45 μm/pixel) slide scanner. The antibodies for immunohistochemical analyses are presented in Table S1 and a flow chart of tumor sampling is shown in Figure S2.

**FIGURE 2:**
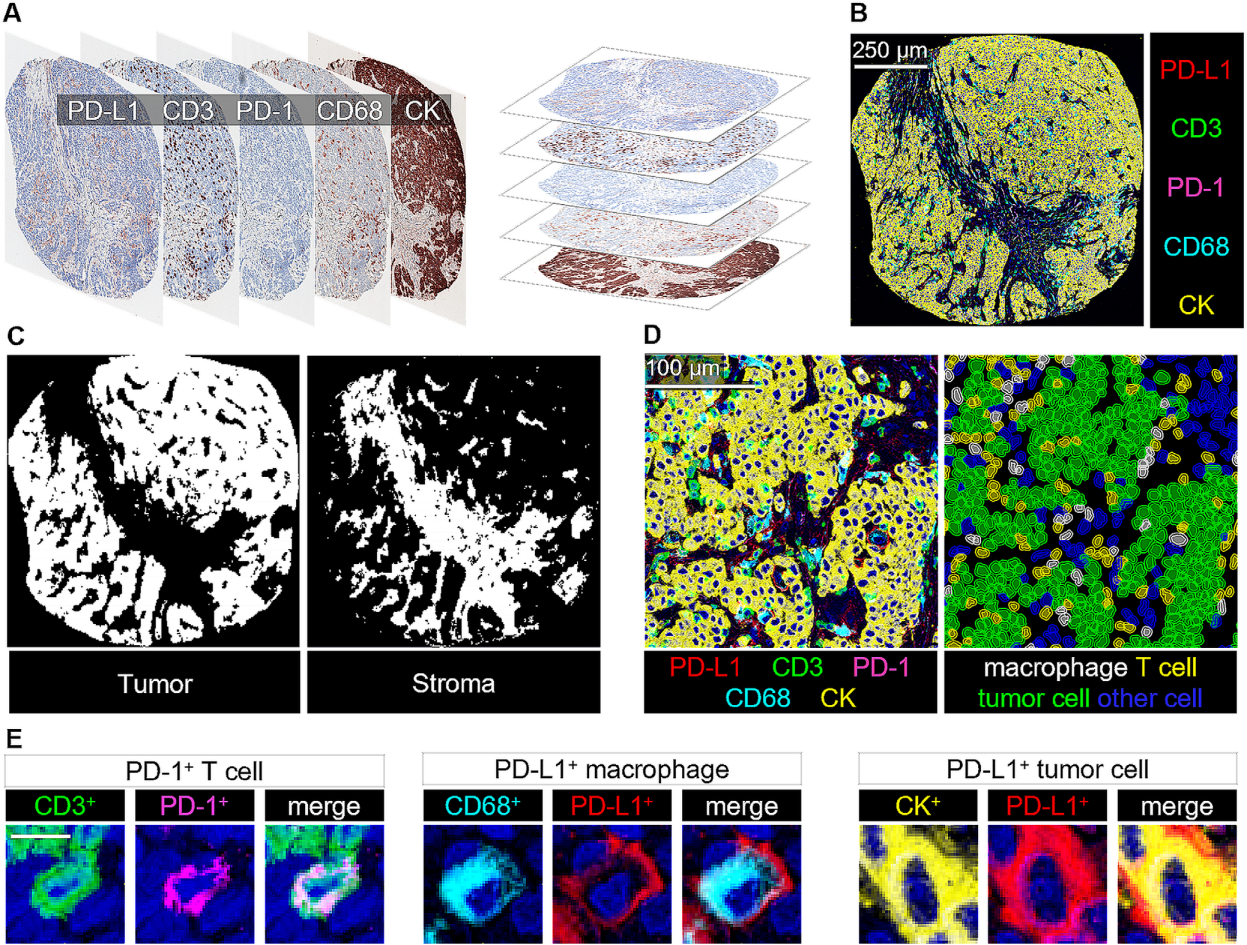
Multiplex immunohistochemistry assay and image analysis. (A) Scanned multiplex immunohistochemistry images and image co‐registration for one example tumor core. The image alignment was based on the similarity of hematoxylin staining in different staining cycles. (B) 5‐plex immunohistochemistry image. (C) Machine learning–based tissue segmentation into tumor epithelial and stromal regions. (D) Multiplex immunohistochemistry image and machine learning–based cell detection and phenotyping into macrophages, T cells, tumor cells, and other cells from the respective tumor region. (E) Examples of three cells with different phenotypes.

To evaluate tumor immune environment, we utilized several detailed methods, including quantitative analysis of H&E‐stained slides (CLR density), spatial (T‐cell proximity score) and density (T‐cell density score) analysis of T lymphocytes in standard immunohistochemistry images, and quantitative analysis of PD‐1 and PD‐L1 expression patterns using multiplex immunohistochemistry (Figures 1 and 2). For immune cell quantification, the TMA sections were scanned and then analyzed by supervised machine learning methods built in the open source bioimage analysis software QuPath (version 0.2.3) [13, 14, 16]. The software was trained to recognize tissue and cell types by manually annotating representative areas or cells. Cells were identified with the *cell detection* function and were phenotyped into T cells, macrophages, tumor cells, and other cells using the *object classifier* function built in QuPath, based on the random forests algorithm. The identification of tissue from the background was done with the random forests *pixel classifier*. T cells were recognized by CD3 or CD8 expression, macrophages were identified through CD68 expression, and tumor cells were identified through keratin expression or characteristic morphology. The remaining cells were classified as other. To evaluate T cell density score, CD3+ and CD8+ cell densities from the tumor center and invasive margin were converted into percentiles and grouped according to the mean of four resulting percentiles [low (0–25), intermediate (> 25–70), and high (> 70–100)] following the principles of the Immunoscore [13, 17]. T‐cell proximity scores were formed according to the G‐cross (G_Tumor:immune cell_) function values at a 20‐μm radius (evaluating the likelihood of any tumor cell in the sample having at least one immune cell of the specified type within a 20‐μm radius) that were converted to percentiles and categorized into three groups (0–25, > 25–70, and > 70–100) similar to T‐cell density score [13]. The T‐cell proximity score analysis is demonstrated in Figure 1b–e. PD‐L1 expression on CD3+ lymphocytes, macrophages, and on tumor cells in multiplex immunohistochemistry images was evaluated as presented earlier [14]. Multiplex immunohistochemistry assay and image analysis is demonstrated in Figure 2. Immune cell densities of all dMMR tumors were distributed into the low, moderate, or high groups according to tertiles (Table S2).

### 2.3 Statistical Analysis

Categorical data were compared using Pearson's chi‐square test. A p‐value < 0.05 was considered statistically significant. Survival times were calculated from the date of surgery to the time of death (cancer related in disease‐specific survival, DSS, or from any reason in overall survival, OS) or the end of follow‐up. Death within 30 days following surgery was considered post‐operative and were excluded from survival analysis (*N* = 9). Univariable and multivariable Cox proportion hazard regression models were used to measure hazard ratio (HR) point estimates and 95% confidence intervals (CIs) for disease‐specific and overall survival. Kaplan–Meier method was used to visualize the estimates of DSS for the main immunological features, and the statistical significance was tested with the log‐rank test. Statistical analyses were performed using IBM SPSS Statistics (version 27.0; SPSS Inc., Chicago, IL, USA).

## 3 Results

Most (68%) of the 171 patients with MMR‐deficient tumors were female. The median age at surgery was 76 (interquartile range, IQR 68–82) years for patients with non‐metastatic tumors and 77 (IQR 65–84) years for patients with metastatic tumors. The median follow‐up time, by using the reverse Kaplan–Meier technique, was 11.5 (95% confidence interval, CI 11.2–13.4) years. The most common MMR protein deficiency pattern was the loss of both MLH1 and PMS2 (*n* = 157, 92%). Concomitant loss of MSH2 and MSH6 was observed in six (5%); concomitant loss of MLH1, PMS2, and MSH6 was observed in three (2%); and isolated loss of MLH1, PMS2, or MSH6 was observed in five (3%) tumors.

Clinicopathological variables were compared between the non‐metastatic and metastatic cases (Table 1). There were no statistically significant differences in age, sex, CCI, lymphovascular invasion, tumor budding, tumor grade, and BRAF mutation between the groups. As expected, higher T, N, and M class and TNM stage were associated with the eventually metastatic disease (*p* < 0.001 for all). Consequently, the metastatic tumor group more often received post‐operative chemotherapy (26% of the non‐metastatic and 63% of the metastatic group, *p* = 0.001). Distal tumor location (*p* = 0.038) and a high tumor necrosis percentage (*p* < 0.001) were more common in the metastatic disease. Although there was no statistically significant difference with T‐cell density scores between the groups, the T‐cell proximity score was generally higher in the non‐metastatic group (*p* = 0.034). Also, CLR density was significantly higher in non‐metastatic tumors (75% with high CLR density in non‐metastatic tumors vs. 40% in metastatic tumors, *p* < 0.001).

**TABLE 1:** Clinicopathological variables for non‐metastatic and metastatic disease in the dMMR CRC cohort.

	Total N of total 171 (% of column)	Non metastatic N of total 136 (% of column)	Metastatic N of total 35 (% of column)	*p*
Age				
< 60	21 (12)	15 (11)	6 (17)	0.523
60–80	95 (56)	78 (57)	17 (49)	
> 80	55 (32)	43 (32)	12 (34)	
Sex				
Male	55 (32)	40 (29)	15 (43)	0.129
Female	116 (68)	96 (71)	20 (57)	
Charlson comorbidity index				
1–2	39 (23)	31 (23)	8 (24)	0.888
3–4	76 (45)	62 (46)	14 (41)	
≥ 5	55 (32)	43 (32)	12 (35)	
Tumor location				
Right hemicolon	146 (85)	120 (88)	26 (74)	0.037
Left hemicolon	25 (15)	16 (12)	9 (26)	
T				
1	10 (6)	10 (7)	0 (0)	< 0.001
2	20 (12)	20 (15)	0 (0)	
3	112 (66)	92 (68)	20 (57)	
4	29 (17)	14 (10)	15 (43)	
N				
0	115 (67)	106 (78)	9 (26)	< 0.001
1	29 (17)	18 (13)	11 (31)	
2	27 (16)	12 (9)	15 (43)	
M				
0	160 (94)	136 (100)	24 (69)	< 0.001
1	11 (6)	0 (0)	11 (31)	
TNM stage				
I	26 (15)	26 (19)	0 (0)	< 0.001
II	85 (50)	80 (59)	5 (14)	
III	49 (29)	30 (22)	19 (54)	
IV	11 (6)	0 (0)	11 (31)	
Lymphovascular invasion				
No	149 (87)	118 (87)	31 (89)	0.776
Yes	22 (13)	18 (13)	4 (11)	
Tumor budding				
0–4/0.785 mm^2^	130 (76)	107 (79)	23 (66)	0.149
5–9/0.785 mm^2^	22 (13)	17 (13)	5 (14)	
10 or more/0.785 mm^2^	19 (11)	12 (9)	7 (20)	
Tumor grade				
Low grade	87 (51)	72 (53)	15 (43)	0.287
High grade	84 (49)	64 (47)	20 (57)	
BRAF mutation				
No	54 (32)	40 (29)	14 (40)	0.229
Yes	117 (68)	96 (71)	21 (60)	
Tumor necrosis				
< 10%	91 (53)	81 (60)	10 (29)	< 0.001
10 to < 40%	58 (34)	44 (32)	14 (40)	
≥ 40%	22 (13)	11 (8)	11 (31)	
Intratumoral stroma				
< 50%	95 (56)	82 (60)	13 (37)	0.014
≥ 50%	76 (44)	54 (40)	22 (63)	
T‐cell density score				
1 (low)	13 (8)	8 (6)	5 (16)	0.160
2 (intermediate)	81 (51)	64 (50)	17 (53)	
3 (high)	65 (41)	55 (43)	10 (31)	
T‐cell proximity score				
1 (low)	10 (6)	7 (6)	3 (10)	0.034
2 (intermediate)	64 (41)	46 (36)	18 (58)	
3 (high)	84 (53)	74 (58)	10 (32)	
Crohn's‐like reaction				
Low	55 (32)	34 (25)	21 (60)	< 0.001
High	116 (68)	102 (75)	14 (40)	
PD‐L1 on tumor cells				
Negative (< 1)	84 (54)	68 (54)	16 (53)	0.874
≥ 1 and < 5	32 (20)	25 (20)	7 (23)	
≥ 5	41 (26)	34 (27)	7 (23	
Lynch syndrome				
No	164 (96)	132 (97)	32 (91)	0.134
Yes	7 (4)	4 (3)	3 (9)	
Adjuvant chemotherapy				
No	113 (66)	100 (74)	13 (37)	< 0.001
Yes	58 (34)	36 (26)	22 (63)	
Postoperative death				
No	162 (95)	129 (95)	33 (94)	0.893
Yes	9 (5)	7 (5)	2 (6)	

*Note:* T‐cell density score was missing from 12 tumors, proximity score was missing from 13 tumors, and Charlson comorbidity index was missing from one patient. PD‐L1 on tumor cells was missing from 14 tumors.

**FIGURE 3:**
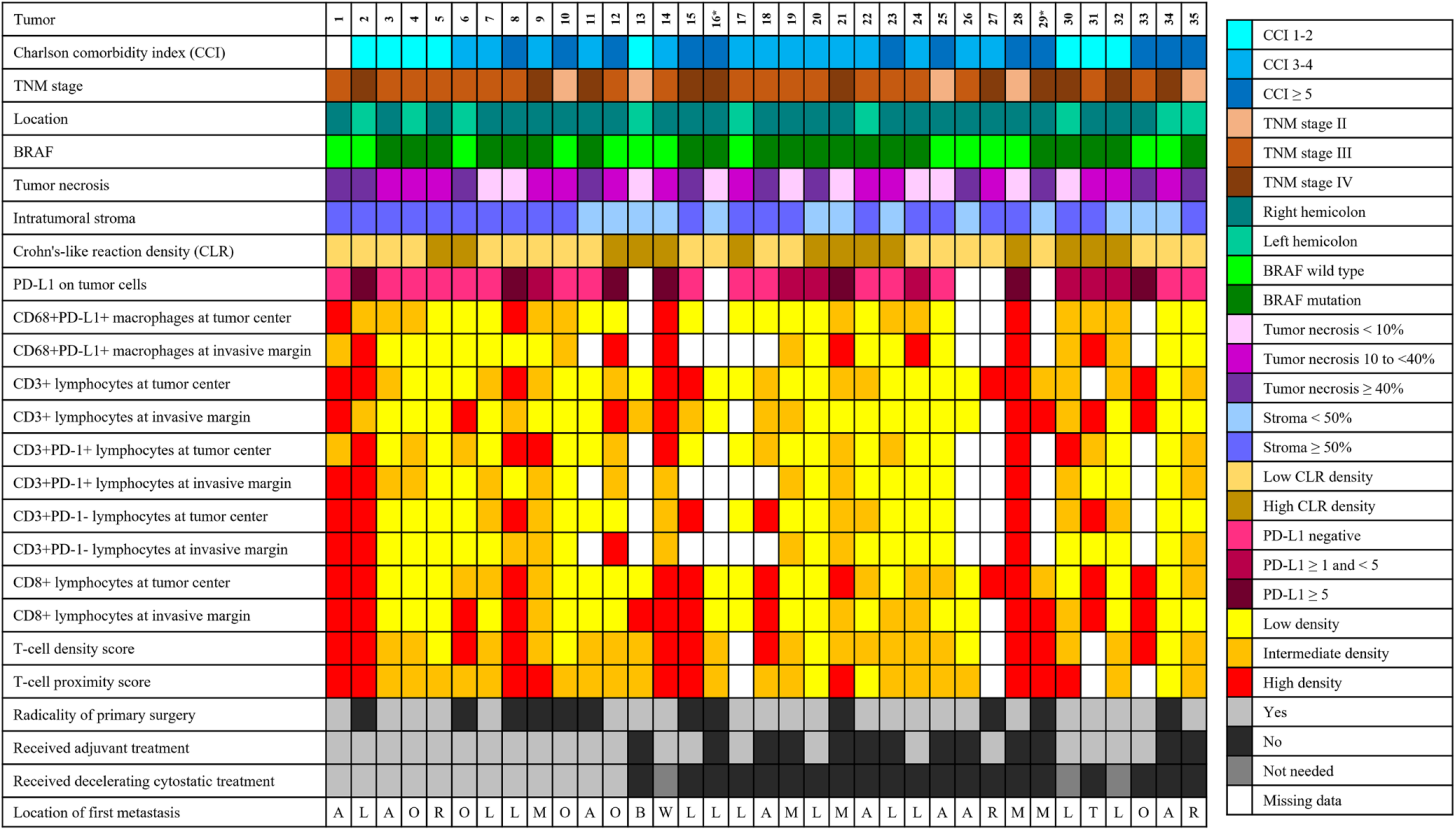
Clinicopathological features and the immune environment of metastatic tumors. The location of metastasis was labeled as follows: A = abdominal cavity meaning peritoneal spread, L = liver, O = origin referring to local recurrence, R = retroperitoneal, M = multiple locations, B = bone, W = wound referring to metastasis at surgical wound, T = thorax referring to lung metastasis. *Postoperative death.

Figure 3 summarizes the histopathological and immune features of the metastatic tumors, and descriptions of the actualized cytostatic treatments and the location of the metastases are presented in Table S3. Eleven tumors (31%) had TNM stage IV at surgery. Three patients had curatively resected metastases followed by standard adjuvant chemotherapy without further evidence of disease recurrence during follow‐up (labeled as not needing decelerating cytostatic treatment). Two patients died postoperatively. A total of 22 (63%) patients received postoperative oncological treatment, 8 of them with unresectable disease or with a positive resection margin (< 1 mm). At the point of identifying a non‐curable disease, 12 (34%) patients were considered fit for cytostatic treatment.

Eleven tumors (31%) had a necrotic component of ≥ 40%, and ten tumors (29%) had less than 10%. High intratumoral stromal component was common in metastatic tumors with 22 (63%) having a stroma percent of ≥ 50%. Only nine tumors (26%) were from the left hemicolon and 21 (61%) had a BRAF mutation.

Histoscore of at least ≥ 1 for the PD‐L1‐positive tumor cells at the tumor center or at the invasive margin was identified in 14 tumors. Eighteen tumors (51%) had at least moderate densities of CD3 lymphocytes at the tumor center and 14 (40%) at the invasive margin, with low densities at both sites in 11 tumors (31%). Eighteen tumors (51%) had at least moderate densities of CD3+PD‐1+ lymphocytes at the tumor center or at the invasive margin, and eight tumors (23%) had low densities in both areas. Eighteen (51%) tumors presented moderate or high densities of CD8+ lymphocytes at the tumor center and 16 (46%) at the invasive margin, with low densities at both sites in 13 tumors (37%). Fifteen (43%) tumors presented at least moderate densities of CD68+PD‐L1+ macrophages at the tumor center and 12 (43%) at the invasive margin, and seven tumors (20%) had low densities in both areas. A high T‐cell density and proximity scores were observed in nine (26%) and in ten tumors (29%), respectively. Fourteen tumors (40%) had a high CLR density.

### 3.1 Survival Analysis

**FIGURE 4:**
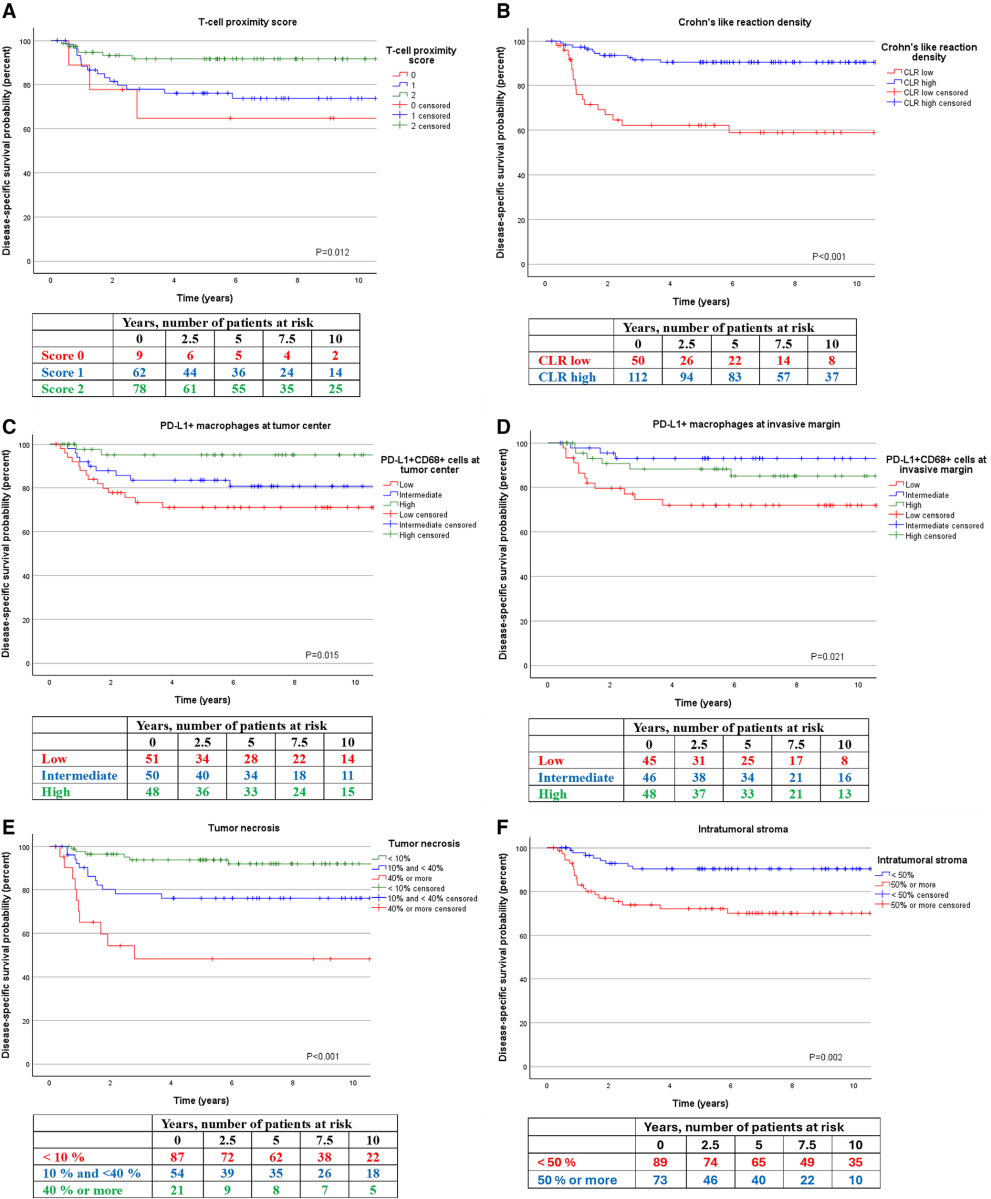
Kaplan–Meier analyses for central tumor microenvironment features. Disease‐specific survival according to (A) T‐cell proximity score, (B) Crohn's‐like reaction density, (C) PD‐L1+ macrophage density at tumor center, (D) PD‐L1+ macrophage density at invasive margin, (E) tumor necrosis, and (F) intratumoral stroma.

Univariable survival analysis for clinicopathological features is shown in Table 2 and in Table S4. TNM stage was a strong prognostic factor for DSS and OS (*p* < 0.001 for both). Also, a high proportion of tumor necrosis and intratumoral stroma were significant prognostic factors for worse DSS (*p* < 0.001 and *p* = 0.004, respectively) as well as for worse OS (*p* = 0.037 and *p* = 0.048, respectively). High CCI was strongly associated with worse OS (*p* < 0.001). High CLR density was prognostic for improved DSS and OS (*p* < 0.001 for both). Higher densities of PD‐L1+ macrophages at the tumor center and invasive margin were prognostic for better DSS (*p* = 0.034 and *p* = 0.035, respectively) but had no significance for OS. Of the different lymphocyte population densities and derived scores, the T‐cell proximity score (*p* = 0.021), CD3+ lymphocytes at the tumor invasive margin (*p* = 0.017), CD3 + PD‐1 negative lymphocytes at the tumor center (*p* = 0.020), CD3 + PD‐1 negative lymphocytes at the invasive margin (*p* = 0.024), and CD8+ lymphocytes at the invasive margin (*p* = 0.049) had a prognostic impact on DSS but had little effect on OS. Kaplan–Meier DSS analyses of the prognostic tumor microenvironment features are shown in Figure 4.

**TABLE 2:** Univariable cox regression analysis for clinicopathological variables.

	*N*	Disease‐specific survival				Overall survival			
		Event	HR	95% CI	*p*	Event	HR	95% CI	*p*
Sex									
Male	52	11	1.34	0.63–2.85	0.442	34	1.37	0.90–2.07	0.143
Female	110	18	1			66	1		
Charlson comorbidity index					0.781				< 0.001
1–2	39	6	1			14	1		
3–4	73	13	1.29	0.49–3.40		49	2.33	1.29–4.23	
≥ 5	49	9	1.45	0.51–4.09		36	3.79	2.02–7.12	
Location									
Right hemicolon	138	21	0.45	0.20–1.01	0.054	86	1.29	0.73–2.28	0.387
Left hemicolon	24	8	1			14	1		
T									
1	10	0		No event	< 0.001	5	1		0.168
2	18	0		No event		11	1.53	0.53–4.42	
3	107	16	1			64	1.58	0.64–3.94	
4	27	13	4.27	2.05–8.91		20	2.54	0.95–6.79	
N									
0	108	8	1		< 0.001	64	1		0.020
1	29	8	4.41	1.65–11.78		18	1.32	0.78–2.23	
2	25	13	9.94	4.09–24.15		18	2.10	1.24–3.57	
TNM stage									
I–II	105	5	1		< 0.001	61	1		< 0.001
III	48	17	9.47	3.49–25.69		30	1.57	1.01–2.44	
IV	9	7	34.38	10.66–110.84		9	8.07	3.81–17.11	
Adjuvant treatment									
No	104	11	1		0.002	68	1		0.412
Yes	58	18	3.13	1.48–6.63		32	0.84	0.55–1.28	
Lymphovascular invasion									
No	143	25	1		0.668	88	1		0.254
Yes	19	4	1.26	0.44–3.63		12	1.43	0.77–2.64	
Tumor budding									
0–4/0.785 mm^2^	124	18	1		0.050	76	1		0.244
5–9/0.785 mm^2^	19	4	1.43	0.48–4.22		11	0.92	0.48–1.74	
10 or more/0.785 mm^2^	19	7	2.99	1.24–7.19		13	1.63	0.90–2.96	
Tumor grade									
Low grade	85	13	1		0.229	54	1		0.298
High grade	77	16	1.57	0.75–3.26		46	1.23	0.83–1.83	
BRAF mutation									
No	52	29	1.63	0.78–3.41	0.193	29	0.66	0.43–1.04	0.71
Yes	110	71	1			71	1		
Tumor necrosis									
< 10%	87	7	1		< 0.001	51	1		0.037
10 to < 40%	54	12	3.03	1.19–7.69		31	0.99	0.63–1.55	
≥ 40%	21	10	8.33	3.16–21.96		18	1.95	1.14–3.35	
Intratumoral stroma									
< 50%	89	9	1		0.004	55	1		0.048
≥ 50%	73	20	3.21	1.46–7.05		45	1.50	1.00–2.23	
Crohn's‐like reaction									
Low	50	18	4.92	2.32–10.43	< 0.001	37	2.05	1.36–3.08	< 0.001
High	112	11	1			63	1		
T‐cell proximity score									
Low	9	3	4.56	1.14–18.26	0.021	5	0.95	0.38–2.39	0.976
Intermediate	62	16	3.47	1.36–8.86		37	1.04	0.68–1.59	
High	78	6	1			49	1		
PD‐L1+ macrophages at the tumor center					0.034				0.652
Low	51	14	6.72	1.53–29.58		34	1.23	0.75–2.03	
Intermediate	50	9	4.05	0.88–18.74		29	1.02	0.61–1.71	
High	48	2	1			29	1		
PD‐L1+ macrophages at the invasive margin					0.035				0.252
Low	45	12	2.24	0.84–5.98		26	1.00	0.60–1.68	
Intermediate	46	3	0.48	0.12–1.93		24	0.67	0.39–1.13	
High	48	6	1			33	1		

*Note:* T‐cell proximity scores were missing from 13 tumors. CD68 (and CD68+PD‐L1+) data were missing from 13 tumor centers and from 23 invasive margin samples. CCI was unknown for one patient.

Abbreviations: CI, confidence interval; HR, hazard ratio; PD‐L1, programmed death‐ligand 1.

Multivariable Cox proportional hazards analyses were performed to further explore the prognostic value of CLR density, PD‐L1+ macrophages at the tumor center and invasive margin, and T‐cell proximity score (Table 3). All statistically significant clinicopathological variables—CCI, TNM stage, tumor necrosis, and intratumoral stroma—were included in the models. All these immune factors proved to have independent prognostic significance for DSS. Most notably, a low T‐cell proximity score had a HR (hazard ratio) of 7.67 (CI 95% 1.42–41.52, *p* = 0.019) and low PD‐L1+ macrophage density at the tumor center had HR of 14.08 (CI 95% 1.72–115.26, *p* = 0.012). CLR density was the only immune factor to independently associate with OS. TNM stage for DSS and OS and CCI for OS remained independently significant factors throughout all analyses. High intratumoral stromal percentage was independently associated with shorter DSS in all analyses except for the model with CLR density.

**TABLE 3:** Multivariable cox regression models for disease‐specific survival and overall survival according to Crohn's‐like reaction density, T‐cell proximity score, and PD‐L1+ macrophage densities at the tumor center and invasive margin.

	Disease‐specific survival		Overall survival	
	HR (95% CI)	*p*	HR (95% CI)	*p*
*Model for Crohn's‐like reaction density*				
Crohn's‐like reaction				
Low	2.71 (1.08–6.79)	0.033	1.63 (1.04–2.54)	0.032
High	1		1	
Charlson comorbidity index				
0–2	1	0.243	1	< 0.001
3	1.45 (0.52–4.01)		2.32 (1.28–4.22)	
≥ 4	2.56 (0.82–8.03)		3.99 (2.11–7.52)	
TNM stage				
I–II	1	< 0.001	1	< 0.001
III	8.66 (3.03–24.77)		1.33 (0.83–2.13)	
IV	27.32 (7.34–101.71)		8.92 (3.97–20.03)	
Tumor necrosis				
< 10%	1	0.022	1	0.558
10 to < 40%	2.50 (0.95–6.56)		0.95 (0.60–1.52)	
≥ 40%	4.87 (1.56–15.19)		1.34 (0.72–2.48)	
Intratumoral stroma				
< 50%	1	0.084	1	0.360
≥ 50%	2.21 (0.90–5.41)		1.23 (0.79–1.92)	
*Model for T‐cell Proximity score*				
T‐cell proximity score				
Low	7.67 (1.42–41.52)	0.019	1.17 (0.44–3.06)	0.430
Intermediate	4.57 (1.48–14.15)		1.36 (0.86–2.17)	
High	1		1	
Charlson comorbidity index				
0–2	1	0.224	1	< 0.001
3	2.30 (0.74–7.20)		2.82 (1.42–5.60)	
≥ 4	2.68 (0.82–8.71)		4.69 (2.31–9.51)	
TNM stage				
I–II	1	< 0.001	1	< 0.001
III	4.12 (1.36–12.43)		1.13 (0.68–1.90)	
IV	39.10 (9.30–164.33)		10.19 (4.34–23.92)	
Tumor necrosis				
< 10%	1	0.046	1	0.360
10 to < 40%	1.58 (1.63–19.02)		0.96 (0.58–1.57)	
≥ 40%	4.06 (1.31–12.57)		1.51 (0.81–2.83)	
Intratumoral stroma				
< 50%	1	0.006	1	0.126
≥ 50%	5.58 (1.63–19.02)		1.46 (0.90–2.37)	
*Model for PD‐L1+ macrophage density at the tumor center*				
PD‐L1+ macrophage density at the CT				
Low	14.08 (1.72–115.26)	0.012	1.50 (0.89–2.54)	0.117
Intermediate	4.70 (0.58–38.09		0.87 (0.50–1.51)	
High	1		1	
Charlson comorbidity index				
0–2	1	0.056	1	< 0.001
3	2.05 (0.67–6.24)		2.28 (1.19–4.37)	
≥ 4	4.52 (1.30–15.70)		4.40 (2.19–8.82)	
TNM stage				
I–II	1	< 0.001	1	< 0.001
III	7.89 (2.42–25.75)		1.27 (0.77–2.10)	
IV	60.42 (12.45–293.16)		10.33 (4.35–24.56)	
Tumor necrosis				
< 10%	1	0.063	1	0.582
10 to < 40%	2.68 (0.96–7.49)		0.99 (0.61–1.62)	
≥ 40%	4.14 (1.19–14.43)		1.38 (0.72–2.63)	
Intratumoral stroma				
< 50%	1	0.003	1	0.113
≥ 50%	7.20 (1.95–26.58)		1.51 (0.91–2.52)	
*Model for PD‐L1+ macrophage density at the invasive margin*				
PD‐L1+ macrophage density at the IM				
Low	2.84 (1.01–7.93)	0.025	1.30 (0.76–2.22)	0.147
Intermediate	0.49 (0.09–2.60)		0.73 (0.42–1.27)	
High	1		1	
Charlson comorbidity index				
0–2	1	0.321	1	0.001
3	1.53 (0.42–5.60)		2.00 (0.98–4.08)	
≥ 4	2.75 (0.70–10.78)		3.61 (1.75–7.44)	
TNM stage				
I–II	1	< 0.001	1	< 0.001
III	5.69 (1.76–18.33)		1.26 (0.76–2.11)	
IV	43.14 (9.00–206.88)		11.36 (4.44–29.05)	
Tumor necrosis				
< 10%	1	0.177	1	0.353
10 to < 40%	2.12 (0.73–6.21)		0.90 (0.54–1.49)	
≥ 40%	3.94 (0.88–17.66)		1.56 (0.75–3.23)	
Intratumoral stroma				
< 50%	1	0.023	1	0.205
≥ 50%	3.83 (1.20–12.29)		1.37 (0.84–2.24)	

## 4 Discussion

We critically evaluated all patients with metastatic dMMR tumors from a large population‐based cohort of CRC patients. We found that the most important prognostic tumor histopathological features in the univariable analyses were TNM stage and proportions of tumor necrosis and intratumoral stroma. Of the immunological factors, CLR density, T‐cell proximity score, and PD‐L1+ macrophages at the tumor center and invasive margin were prognostic in univariable analyses and remained so also in the multivariable models, when adjusted for CCI, TNM stage, tumor necrosis, and intratumoral stroma. Only 12 patients (34% of all metastatic dMMR, 7% of all dMMR tumors, and 0.9% from the total study population) were fit enough to initiate conventional cytostatic treatments at the onset of the non‐curable metastatic disease. Also, many of the metastatic tumors presented features that may suggest a reduced benefit from PD‐1 blockade therapy.

Encouraging results from a phase III KEYNOTE‐177 trial showed significant improvement in progression‐free survival in metastatic dMMR CRC for first‐line treatment with PD‐1 blockade pembrolizumab versus chemotherapy (16.5 months vs. 8.2 months; hazard ratio 0.60; *p* = 0.0002). Also, the response rate to treatment was higher with pembrolizumab (45% vs. 33%) [18]. Another anti‐PD‐1 agent, nivolumab, demonstrated an objective response rate of 31% and median progression‐free survival of 14.3 months for metastatic dMMR CRC with prior treatments [19]. The combination of nivolumab with ipilimumab, a cytotoxic T lymphocyte antigen‐4 (CTLA‐4) inhibitor, had an improved objective response rate of 65% and disease control rate of 81% at 50.9 months [20]. As demonstrated in these studies, the response to treatment is not self‐evident. MSI, PD‐L1 expression on tumor cells, and a high tumor mutation burden are the best described biomarkers for predicting the efficacy of the PD‐1/PD‐L1 pathway blockade. In addition, a high number of tumor‐infiltrating lymphocytes, especially CD8+ cytotoxic T lymphocytes and B lymphocytes, and PD‐L1 expression on peritumoral immune cells, mainly macrophages, are associated with treatment response [21]. As MSI tumors are often hypermutated with an abundant neoantigen load, high lymphocyte infiltration is common [9].

In our study, T‐cell proximity and density scores were high in 53% and 41% of the MMR deficient tumors, respectively, and low scores were not common even in metastatic tumors. In fact, the beneficial impact on survival was evident mainly in highest T‐cell proximity scores. High CD3+PD‐1‐ densities at tumor center and at invasive margin showed survival benefit unlike the high CD3+PD‐1+ densities suggesting immune exhaustion in PD‐1+ cells. Eighteen metastatic tumors (51%) presented high to intermediate densities of CD3+PD‐1+ cells with the potential of regain of function with PD‐1 blockade therapy [22]. However, 13 tumors (37%) presented low densities of CD8+ lymphocytes, seven of those with concomitant PD‐L1 expression at tumor microenvironment and therefore potentially lacking the important cytotoxic T‐cell activity even if the inhibitory PD‐1/PD‐L1 pathway would be blocked. Additionally, 11 (31%) of the metastatic tumors did not have significant expression of PD‐L1 on macrophages or on tumor cells, seven of those additionally had low CD3+PD‐1+ lymphocyte densities, and four lacked also CD8+ lymphocytes suggesting poor response for possible PD‐1 blockade therapy.

Tumor necrosis is a common feature in the tumor microenvironment and a sign of more aggressive tumor growth that is associated with higher tumor stage, poor differentiation, and poor prognosis in CRC [23]. In addition to potentially reduced access of therapeutic agents at the hypoxic tumor site, hypoxic conditions support cancer immune evasion in several ways, for example, by inducing the expression of immune checkpoint molecules and promoting immunosuppressive cells while inhibiting tumor‐infiltrating lymphocytes [24]. In our study, tumor necrosis was significantly more common in metastatic tumors, as 10 patients (71%) presented with tumor necrosis of 10% or more and 11 (31%) patients with 40% or more. Intratumoral stroma contributes to virtually all stages of cancer progression and acts as a barrier preventing anti‐tumoral immune activation; it is associated with resistance against anticancer therapiesand, in high proportions, predicts poor survival [25]. In concordance with this, we found that high intratumoral stroma was more common in metastatic tumors (63% vs. 40%) and was associated with worse DSS and OS. Up to 91% of the metastatic tumors had significant proportions of intratumoral necrosis and/or stroma and therefore might have been prone to treatment resistance.

Before initiating treatments, it is necessary to consider that immune checkpoint inhibitors may cause serious adverse effects. Within immune checkpoint inhibitor phase 2 and 3 clinical trials, the pooled incidence of all adverse events ranged from 66% to 87%, while serious or life‐threatening adverse events ranged from 14% to 29% [26]. Adverse events can occur in a wide range of presentations. However, organ‐specific manifestations (especially cardiac, liver, and pulmonary) are the most important due to higher rates of associated mortality [27]. Sporadic dMMR CRC patients are usually older and therefore have more comorbidities. Aging also gradually deteriorates the immune system, and therefore, the response to immune‐oncologic therapies may be weaker in the elderly [28]. In our study, the median age of dMMR CRC patients during surgery was 77 years. Only 39 (23%) of our patients had a CCI of 2 or less, and 55 (32%) had a CCI of 5 or more. From the metastatic group, we found that 12 patients had no significant comorbidities (other than a high age in two) and, although 22 (63%) received adjuvant treatments postoperatively, only 12 (34%) patients were fit enough to initiate oncological treatments at the onset of the metastatic disease.

Crohn's‐like reaction (CLR) denotes peritumoral lymphoid aggregates that provide a local site for tumor antigen presentation for dendritic cells, leading to the activation of T and B lymphocytes. CLR is associated with an improved prognosis and is frequently present in MSI tumors [29]. Here, CLR density was significantly higher in non‐metastatic tumors and proved to be the only independent prognostic immune feature of the tumor microenvironment to impact DSS and OS. Fourteen metastatic tumors (40%) showed high CLR densities. Despite its evidently important function in antitumoral immunity, the role of CLR in predicting immunotherapy efficacy is unknown and requires further studies [29].

As a limitation we evaluated the tumor microenvironment only from the primary tumors, but the differences of the immune contexture between primary tumors and metastases may also have an influence on treatment efficacy. Unfortunately, there were no samples from metastatic sites available to be compared with primary tumors. Previous studies comprising of mostly MMR proficient tumors show that immune cell infiltration and PD‐L1 expression seems to be higher in liver and lung metastases compared to matched primary CRCs [30, 31, 32]. Also, recent preliminary results presented in the congress of European Society for Medical Oncology suggests that tumor mutation burden, neoantigen load, and PD‐L1 expression are similar in MSI primary tumors and in metastases explaining the benefits of immune therapy seen across different metastatic sites [33]. Some evidence suggests that resistance to PD‐1 blockade monotherapy in metastatic dMMR CRC is related to lower densities of CD8+ T cells and monocytes in the primary tumors and, that resistant tumors had high expression of IL‐1β with accumulation of immunosuppressive myeloid‐derived suppressor cells [34]. Another study found that high overall CD8+lymphocyte density, high number of CD8+ T‐cells expressing PD‐1, and tumor‐infiltrating immune cells with a Th1 phenotype were associated with tumors responsive to immunotherapy [35]. Considering these findings, some, although reserved, conclusions of the usability of PD‐1 blockade therapy could be made from samples from primary tumors. Furthermore, as up to date guidelines recommend that pembrolizumab should be offered as first‐line therapy to patients with MSI‐high or deficient mismatch repair mCRC [36, 37], our evaluation of the surgically incurable tumors provide relevant additional data of the microenvironment of advanced dMMR CRC.

This study also has other limitations because it utilizes a retrospective series of CRC (although large and representative) to evaluate the tumor microenvironment of patients with non‐metastatic and metastatic dMMR CRC, focusing on the characteristics of metastatic cases that could potentially influence response to immune checkpoint therapy, which was not available during their treatment period. The total number of dMMR tumors and cancer‐related terminal events are relatively small, so the prognostic conclusions from survival analyses should be considered suggestive. Although conventional histopathological data and Crohn's‐like reaction were evaluated from whole‐tissue sections, the analyses of individual immune cells were based on tissue microarrays, which allow sampling from only a small part of the tumor and may not fully represent the entire tumor. However, we analyzed multiple cores from each tumor, and the cores were selected from different sites to more accurately represent average immune cell infiltrates. Furthermore, the temporal changes in the tumor microenvironment at different stages of tumorigenesis are not captured in a surgical excision sample in a metastatic situation where the residual tumor continues to evolve [38]. We did not have data on *KRAS* and *NRAS* mutations, which is an important limitation. In addition, we did not analyze several potentially relevant immune cell populations, such as B‐lymphocytes or double‐negative T‐cells, nor did we assess the expression levels of several key immune checkpoint proteins, such as CTLA‐4 (cytotoxic T‐lymphocyte‐associated protein 4), TIM‐3 (T‐cell immunoglobulin and mucin‐domain containing‐3), TIGIT (T‐cell immunoreceptor with Ig and ITIM domains), or LAG‐3 (lymphocyte activation gene 3). These factors could be important when evaluating the potential of immunotherapies [39, 40, 41]. It should be noted that other cancer immune escape mechanisms, such as interferon gamma unresponsiveness, may also weaken the treatment efficacy [42]. As a strength, the study population was consecutive and population‐based, with thorough clinicopathological data and complete follow‐up information.

## 5 Conclusions

In conclusion, metastatic dMMR CRC comprises less than 3% of all CRCs. As dMMR CRC patients are generally older, with often significant comorbidities, only a limited portion of patients with metastatic disease seem to end up in oncological treatments. Several immune factors had an independent prognostic impact and could have potential in estimating tumor behavior. Tumor necrosis and intratumoral stroma are common in metastatic tumors and may cause resistance to oncological treatments. Based on the characteristics of the primary tumor immune microenvironment, 12 metastatic tumors (34%) had features suggesting potential response to PD‐1 blockade therapy, and four tumors (11%) were immunologically cold, while the rest either had T cells without active checkpoint molecule expression or vice versa. Accordingly, it is probable that the response to PD‐1 blockade therapy would have been achievable in only some of the metastatic tumors in this unselected and population‐based series of CRC patients.

## Author Contributions

**Erkki‐Ville Wirta:** conceptualization (equal), data curation (equal), formal analysis (lead), funding acquisition (equal), investigation (equal), methodology (equal), visualization (equal), writing – original draft (lead), writing – review and editing (lead). **Hanna Elomaa:** data curation (equal), formal analysis (equal), investigation (lead), methodology (lead), visualization (equal), writing – review and editing (equal). **Jukka‐Pekka Mecklin:** conceptualization (equal), data curation (equal), funding acquisition (lead), investigation (equal), resources (lead), supervision (lead), writing – original draft (supporting), writing – review and editing (equal). **Toni T. Seppälä:** data curation (equal), investigation (equal), writing – review and editing (equal). **Marja Hyöty:** investigation (equal), supervision (equal), writing – review and editing (equal). **Jan Böhm:** data curation (equal), investigation (equal), resources (equal), writing – review and editing (equal). **Maarit Ahtiainen:** data curation (equal), investigation (equal), writing – review and editing (equal). **Juha P. Väyrynen:** conceptualization (equal), data curation (equal), formal analysis (equal), funding acquisition (equal), investigation (lead), methodology (lead), resources (equal), supervision (lead), visualization (equal), writing – original draft (supporting), writing – review and editing (equal).

## Ethics Statement

The study was conducted according to the guidelines of the Declaration of Helsinki and approved by both the hospital administration and the ethics board (Dnro13U/2011, 1/2016, and 8/2020) and by the National Supervisory Authority for Welfare and Health (Valvira). The need to obtain informed consent from the study patients was waived (Valvira Dnro 3916/06.01.03.01/2016).

## Conflicts of Interest

T.T.S. reports a consultation fee from Amgen Finland, being a co‐owner and CEO of Healthfund Finland Ltd., and sitting on the Clinical Advisory Board of LS Cancer Diag Ltd. Otherwise, the authors declare no conflicts of interest.

## Supporting information


**Figure S1.** Receiver operating characteristic curve for Crohn’s‐like reaction density.

**Figure S2.** A flow chart of tumor sampling.

**Table S1.** Antibodies used for immunohistochemistry.

**Table S2.** Immune cell tertiles in non‐metastatic and metastatic tumors.

**Table S3.** Actualized oncological treatments and the location of the metastases.

**Table S4.** Univariable Cox proportional hazards models for immune factors.

## Data Availability

The datasets generated and/or analyzed during this study are not publicly available due to Finnish laws on privacy protection. The sharing of data will require approval from relevant ethics committees and/or biobanks. Further information, including the procedures to obtain and access data from Finnish biobanks, is described at https://finbb.fi/en/fingenious‐service.
