# Supplementary figures and images for: Prognostic Features and Potential for Immune Therapy in Metastatic Mismatch Repair‐Deficient Colorectal Cancer: A Retrospective Analysis of a Large Consecutive Population‐Based Patient Series
Source: Cancer Med. 2025 Jan 9;14(1):e70555. doi: 10.1002/cam4.70555 (PMC11714176; doi:10.1002/cam4.70555)

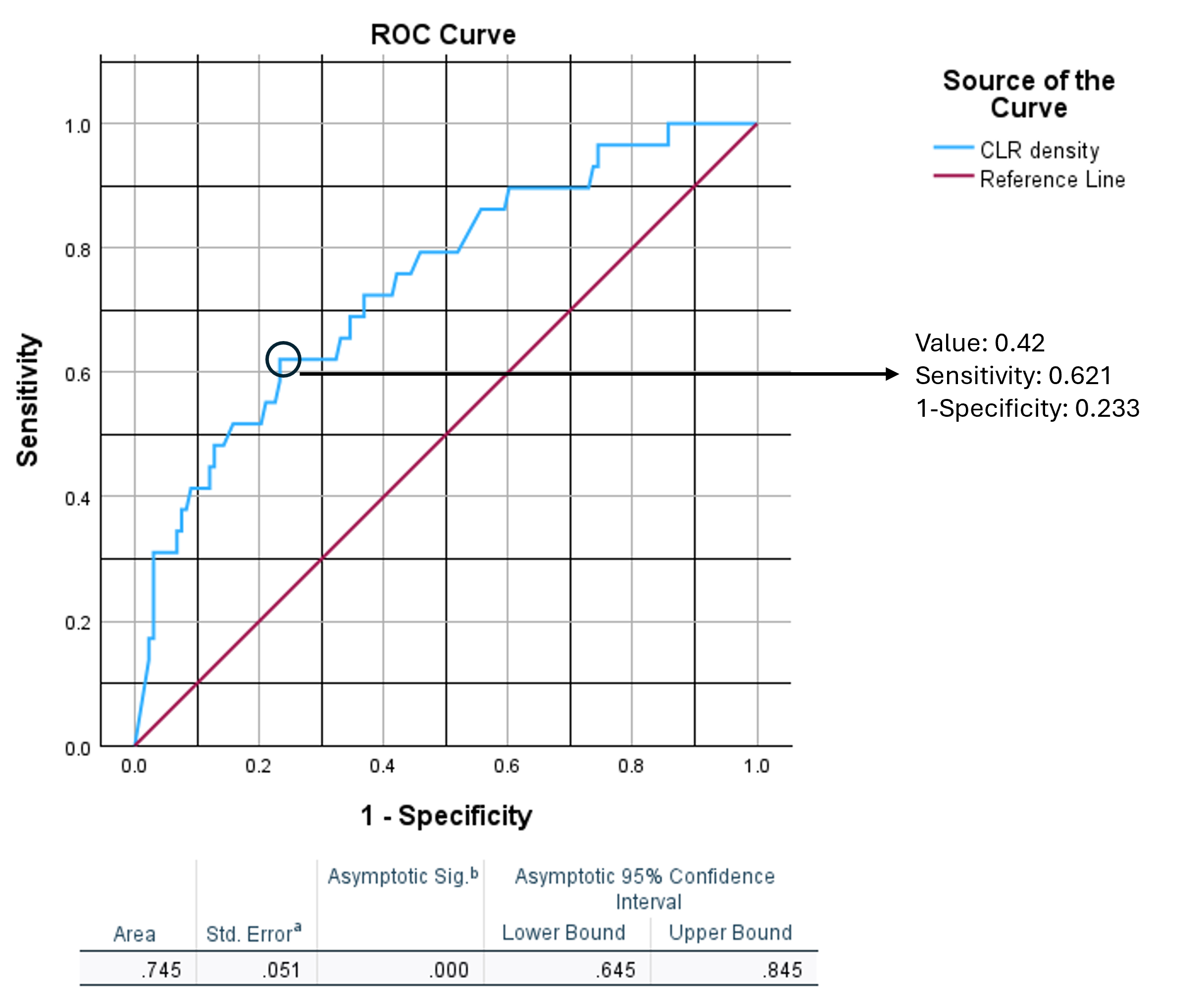

Supplement: Supplementary file 1 — Figure S1. Receiver operating characteristic curve for Crohn’s‐like reaction density. [file CAM4-14-e70555-s004.tif]

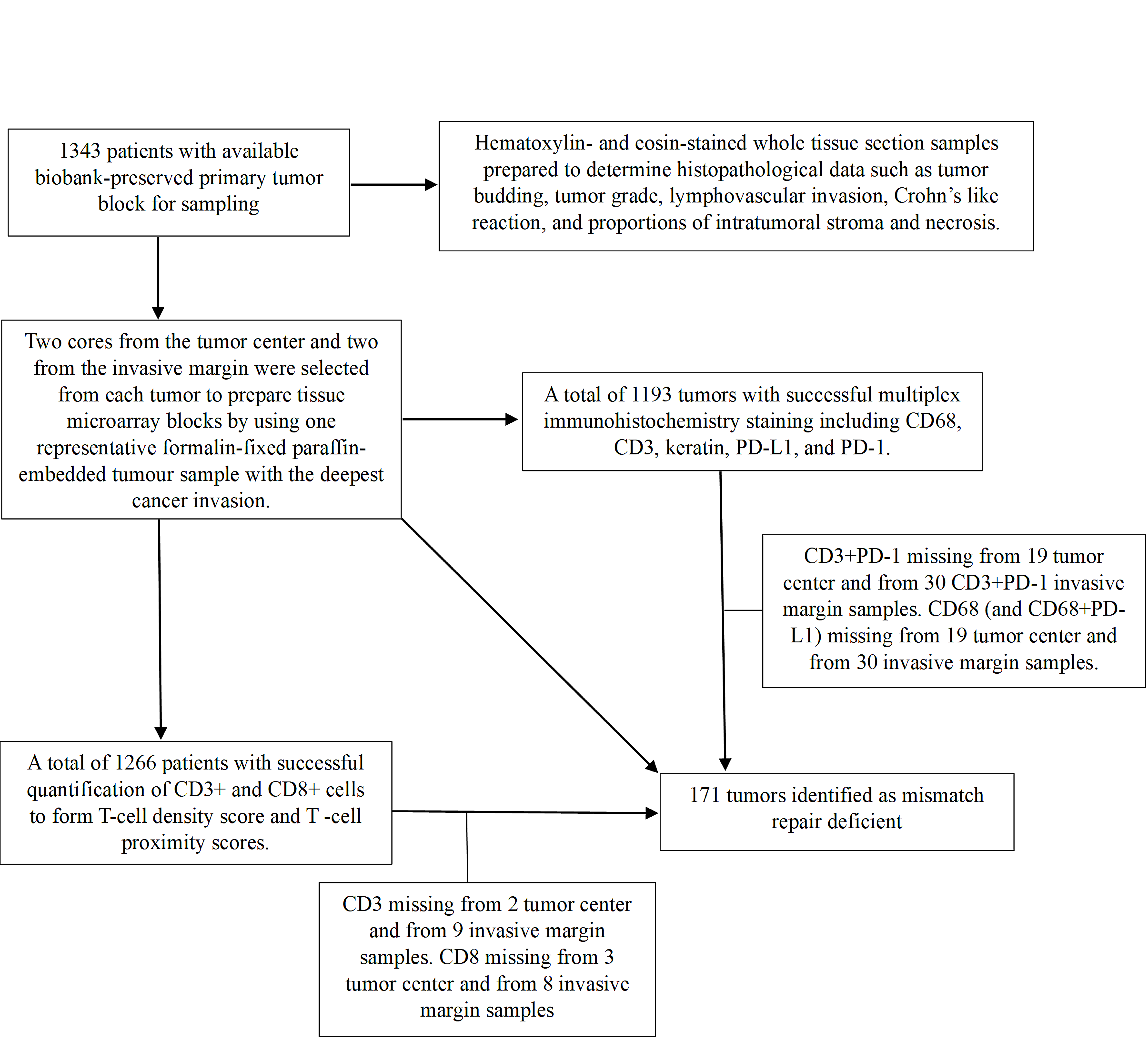

Supplement: Supplementary file 2 — Figure S2. A flow chart of tumor sampling. [file CAM4-14-e70555-s006.tif]
