# Supplementary material for: Prognostic Features and Potential for Immune Therapy in Metastatic Mismatch Repair‐Deficient Colorectal Cancer: A Retrospective Analysis of a Large Consecutive Population‐Based Patient Series
Source: Cancer Med. 2025 Jan 9;14(1):e70555. doi: 10.1002/cam4.70555 (PMC11714176; doi:10.1002/cam4.70555)
Supplement: Supplementary file 3 — Table S1. Antibodies used for immunohistochemistry. [file CAM4-14-e70555-s005.docx]

| **Antigen** | **Target** | **Manufacturer** | **Clone** | **Code** |
| --- | --- | --- | --- | --- |
| CD3 | T cell | Leica Biosystems | LN10 | PA055 |
| CD8 | Cytotoxic T cell | Thermo Scientific | SP16 | RM-9116 |
| MLH1 | MLH1 protein | Novocastra | ES05 | NCL-L-MLH1 |
| MSH2 | MSH2 protein | Calbiochem | FE11 | NA27 |
| MSH6 | MSH6 protein | Epitomics | EP49 | AC-0047 EU |
| PMS2 | PMS2 protein | BD-Pharmingen | A16-4 | 5564151 |
| BRAF^V600E^ | BRAF^V600E^ mutated protein | Spring Bioscience | VE1 | E19292 |
| CD274 (PD-L1) | CD274 protein | Cell Signaling | E1L3N | 13684S |
| PDCD1 (PD-1) | PDCD1 protein | Abcam | SP269 | ab227681 |
| CD68 | Macrophage | Biolegend | KP1 | 916104 |
| KRT | Epithelial cell | BioSite Histo | BS5 | BSH-7124-1 |

**Supplementary table 1. Antibodies used for immunohistochemistry**
