# Supplementary material for: Prognostic Features and Potential for Immune Therapy in Metastatic Mismatch Repair‐Deficient Colorectal Cancer: A Retrospective Analysis of a Large Consecutive Population‐Based Patient Series
Source: Cancer Med. 2025 Jan 9;14(1):e70555. doi: 10.1002/cam4.70555 (PMC11714176; doi:10.1002/cam4.70555)
Supplement: Supplementary file 4 — Table S2. Immune cell tertiles in non‐metastatic and metastatic tumors. [file CAM4-14-e70555-s002.docx]

**Supplementary table 2. Immune cell tertiles in non-metastatic and metastatic tumors**

|  | **Total**  N of total 171  (% of column) | **Non metastatic** N of total 136  (% of column) | **Metastatic**  N of total 35  (% of column) | **P** |
| --- | --- | --- | --- | --- |
| **CD3 overall cell density at tumor center**  T1  T2  T3 | 57 (34)  56 (33)  57 (34) | 42 (31)  45 (33)  49 (36) | 15 (44)  11 (32)  8 (24) | 0.260 |
| **CD3 overall cell density at invasive margin**  T1  T2  T3 | 53 (33)  55 (34)  55 (34) | 35 (27)  48 (37)  47 (36) | 18 (55)  7 (21)  8 (24) | 0.010 |
| **CD8 overall cell density at tumor center**  T1  T2  T3 | 56 (33)  56 (33)  57 (34) | 40 (30)  48 (36)  46 (34) | 16 (46)  8 (23)  11 (31) | 0.167 |
| **CD8 overall cell density at invasive margin**  T1  T2  T3 | 55 (34)  54 (33)  55 (34) | 38 (29)  49 (38)  43 (33) | 17 (50)  5 (15)  12 (35) | 0.020 |
| **CD3+PD-1+ cell density at tumor center**  T1  T2  T3 | 51 (34)  51 (34)  50 (33) | 37 (30)  42 (34)  44 (36) | 14 (48)  9 (31)  6 (21) | 0.135 |
| **CD3+PD-1- cell density at tumor center**  T1  T2  T3 | 51 (34)  51 (34)  50 (33) | 36 (29)  44 (36)  43 (35) | 15 (52)  7 (24)  7 (24) | 0.070 |
| **CD3+PD-1+ cell density at invasive margin**  T1  T2  T3 | 46 (33)  48 (34)  47 (33) | 34 (29)  38 (33)  44 (38) | 12 (48)  10 (40)  3 (12) | 0.036 |
| **CD3+PD-1- cell density at invasive margin**  T1  T2  T3 | 47 (33)  46 (33)  48 (34) | 32 (28)  40 (35)  44 (38) | 15 (60)  6 (24)  4 (16) | 0.006 |
| **CD68 overall cell density at tumor center**  T1  T2  T3 | 51 (34)  51 (34)  50 (33) | 38 (31)  42 (34)  43 (35) | 13 (45)  9 (31)  7 (24) | 0.323 |
| **CD68 overall cell density at invasive margin**  T1  T2  T3 | 47 (33)  46 (33)  48 (34) | 36 (31)  39 (34)  41 (35) | 11 (44)  7 (28)  7 (28) | 0.458 |
| **CD68+PD-L1+ cell density at tumor center**  T1  T2  T3 | 51 (34)  51 (34)  50 (33) | 37 (30)  40 (33)  46 (37) | 14 (48)  11 (38)  4 (14) | 0.039 |
| **CD68+PD-L1- cell density at tumor center**  T1  T2  T3 | 51 (34)  50 (33)  51 (34) | 38 (31)  42 (34)  43 (35) | 13 (45)  8 (28)  8 (28) | 0.360 |
| **CD68+PD-L1+ cell density at invasive margin**  T1  T2  T3 | 46 (33)  47 (33)  48 (34) | 33 (28)  42 (36)  41 (35) | 13 (52)  5 (20)  7 (28) | 0.066 |
| **CD68+PD-L1- cell density at invasive margin**  T1  T2  T3 | 46 (33)  47 (33)  48 (34) | 36 (31)  38 (33)  42 (36) | 10 (40)  9 (36)  6 (24) | 0.479 |
| CD3 data were missing from two (and from 19 CD3+PD-1) tumor center and from nine (and from 30 CD3+PD-1+) invasive margin samples. CD8 data were missing from three tumor center and from eight invasive margin samples. CD68 (and CD68+PD-L1+) data were missing from 19 tumor center and from 30 invasive margin samples. | | | | |
