# Supplementary material for: Prognostic Features and Potential for Immune Therapy in Metastatic Mismatch Repair‐Deficient Colorectal Cancer: A Retrospective Analysis of a Large Consecutive Population‐Based Patient Series
Source: Cancer Med. 2025 Jan 9;14(1):e70555. doi: 10.1002/cam4.70555 (PMC11714176; doi:10.1002/cam4.70555)
Supplement: Supplementary file 5 — Table S3. Actualized oncological treatments and the location of the metastases. [file CAM4-14-e70555-s003.docx]

**Supplementary table 3. Actualized oncological treatments and the location of the metastases**

| **Cytostatic treatment** | **After surgery**  N=35 (% of column) | **After recurrence or progression**  N=30 (% of column) | **After further progression**  N=8 (% of column) |
| --- | --- | --- | --- |
| Fluorouracil or single capecitabine | 11 (31%) | 1 (3%) | 1 (13%) |
| CAPOX | 4 (11%) | 0 (0%) | 0 (0%) |
| FOLFOX or FOLFIRI | 3 (9%) | 1 (3%) | 0 (0%) |
| FOLFOX or FOLFIRI with bevacizumab | 2 (6%) | 2 (7%) | 0 (0%) |
| Other or unspecified | 2 (6%) | 4 (13%) | 1 (13%) |
| No cytostatic treatments due to poor overall health | 10 (29%) | 22 (73%) | 6 (75%) |
| Treatment considered unnecessary | 1 (3%) | 0 (0%) | 0 (0%) |
| Postoperative death | 2 (6%) | N/A | N/A |
| **Location of first metastasis:**  Liver  Lung  Peritoneal carcinosis  Retroperitoneal  Local recurrence  Bone  Surgical wound  Multiple locations | 11 (31%)  1 (3 %)  8 (23%)  3 (9%)  5 (14%)  1 (3%)  1 (3%)  5 (14%) | | |
| Abbrevations: CAPOX: capecitabine and oxaliplatin; FOLFOX: folinic acid, fluorouracil, and oxaliplatin; FOLFIRI: folinic acid, fluorouracil and irinotecan.  Three patients underwent R0 resection of the metastasis without further recurrence. One patient received preoperative FOLFOX before resection of a metastasis, otherwise none of the patients in the metastatic group received neoadjuvant treatment. Fluorouracil/single capesitabine treatment after surgery was ceased early for four patients and FOLFOX/FOLFIRI with bevacizumab was ceased for one patient because of declining health. | | | |
