# Supplementary material for: Prognostic Features and Potential for Immune Therapy in Metastatic Mismatch Repair‐Deficient Colorectal Cancer: A Retrospective Analysis of a Large Consecutive Population‐Based Patient Series
Source: Cancer Med. 2025 Jan 9;14(1):e70555. doi: 10.1002/cam4.70555 (PMC11714176; doi:10.1002/cam4.70555)
Supplement: Supplementary file 6 — Table S4. Univariable Cox proportional hazards models for immune factors. [file CAM4-14-e70555-s001.docx]

**Supplementary table 4. Univariable Cox proportional hazards models for immune factors**

|  |  | Disease-specific survival | | | | Overall survival | | | |
| --- | --- | --- | --- | --- | --- | --- | --- | --- | --- |
|  | N | Event | HR | 95 % CI | P | Event | HR | 95 % CI | P |
| **PD-L1 on tumor cells at TC:**  < 1  ≥ 1 and <5  ≥ 5 | 98  30  22 | 21  4  1 | 4.78  3.12  1 | 0.64–35.55  0.35–27.91 | 0.251 | 60  21  12 | 1.46  1.76  1 | 0.78–2.74  0.86–3.60 | 0.299 |
| **PD-L1+ macrophages at TC:**  Low  Intermediate  High | 51  50  48 | 14  9  2 | 6.72  4.05  1 | 1.53–29.58  0.88–18.74 | 0.034 | 34  29  29 | 1.23  1.02  1 | 0.75–2.03  0.61–1.71 | 0.652 |
| **PD-L1- macrophages at TC:**  Low  Intermediate  High | 50  50  49 | 12  7  6 | 1.94  1.11  1 | 0.73–5.17  0.37–3.29 | 0.310 | 34  30  28 | 1.15  1.15  1 | 0.69–1.90  0.69–1.93 | 0.832 |
| **PD-L1+ macrophages at IM:**  Low  Intermediate  High | 45  46  48 | 12  3  6 | 2.24  0.48  1 | 0.84–5.98  0.12–1.93 | 0.035 | 26  24  33 | 1.00  0.67  1 | 0.60–1.68  0.39–1.13 | 0.252 |
| **PD-L1- macrophages at IM:**  Low  Intermediate  High | 46  47  46 | 8  7  6 | 1.42  1.16  1 | 0.49–4.08  0.39–3.46 | 0.809 | 27  28  28 | 0.95  0.95  1 | 0.56–1.61  0.56–1.60 | 0.975 |
| **T-cell density score:**  Low  Intermediate  High | 13  77  60 | 5  14  7 | 3.17  1.47  1 | 1.01–10.01  0.59–3.63 | 0.139 | 9  46  37 | 1.27  0.89  1 | 0.61–2.63  0.57–1.37 | 0.591 |
| **T-cell proximity score:**  Low  Intermediate  High | 9  62  78 | 3  16  6 | 4.56  3.47  1 | 1.14–18.26  1.36–8.86 | 0.021 | 5  37  49 | 0.95  1.04  1 | 0.38–2.39  0.68–1.59 | 0.976 |
| **CD3+ lymphocytes at TC:**  Low  Intermediate  High | 54  54  53 | 14  8  6 | 2.28  1.16  1 | 0.88–5.94  0.40–3.34 | 0.141 | 38  29  32 | 1.34  0.72  1 | 0.84–2.14  0.43–1.19 | 0.043 |
| **CD3+ lymphocytes at IM:**  Low  Intermediate  High | 51  53  50 | 16  5  6 | 2.68  0.77  1 | 1.05–6.86  0.23–2.52 | 0.017 | 32  32  30 | 1.15  0.95  1 | 0.70–1.89  0.57–1.56 | 0.734 |
| **CD3+PD-1+ lymphocytes at TC:**  Low  Intermediate  High | 51  49  49 | 13  9  3 | 4.04  2.77  1 | 1.15–14.17  0.75–10.22 | 0.090 | 33  30  29 | 1.07  0.91  1 | 0.65–1.77  0.54–1.51 | 0.796 |
| **CD3+PD-1- lymphocytes at TC:**  Low  Intermediate  High | 51  50  48 | 15  4  6 | 2.30  0.55  1 | 0.89–5.92  0.15–1.93 | 0.020 | 33  31  28 | 1.12  0.86  1 | 0.68–1.86  0.52–1.44 | 0.578 |
| **CD3+PD-1+ lymphocytes at IM:**  Low  Intermediate  High | 45  48  46 | 10  8  3 | 3.53  2.70  1 | 0.97–12.81  0.72–10.17 | 0.160 | 27  28  28 | 0.95  0.94  1 | 0.56–1.61  0.56–1.59 | 0.971 |
| **CD3+PD-1- lymphocytes at IM:**  Low  Intermediate  High | 46  46  47 | 13  4  4 | 3.42  1.01  1 | 1.12–10.49  0.25–4.03 | 0.024 | 25  29  29 | 0.88  0.91  1 | 0.51–1.50  0.54–1.53 | 0.882 |
| **CD8+ lymphocytes at TC:**  Low  Intermediate  High | 54  51  55 | 13  7  9 | 1.41  0.77  1 | 0.60–3.29  0.29–2.05 | 0.411 | 35  32  32 | 1.09  0.96  1 | 0.67–1.76  0.59–1.57 | 0.880 |
| **CD8+ lymphocytes at IM:**  Low  Intermediate  High | 53  50  52 | 15  4  9 | 1.52  0.39  1 | 0.67–3.48  0.12–1.26 | 0.049 | 33  26  36 | 0.85  0.65  1 | 0.53–1.37  0.39–1.08 | 0.252 |
| Abbreviations: PD-L1: programmed death-ligand 1; PD-1: Programmed cell death protein 1; TC: tumor center; IM: invasive margin; HR: hazard ratio; CI: confidence interval.  CD3 data were missing from two (and from 13 CD3+PD-1) tumor center and from nine (and from 23 CD3+PD-1+) invasive margin samples. CD8 data were missing from two tumor center and from seven invasive margin samples. T-cell density score was missing from 12 and proximity score was missing from 13 tumors. CD68 (and CD68+PD-L1+) data were missing from 13 tumor center and from 23 invasive margin samples. CCI was unknown from one patient. | | | | | | | | | |
